# Vaccine-induced immune thrombotic thrombocytopenia: Updates in pathobiology and diagnosis

**DOI:** 10.3389/fcvm.2022.1040196

**Published:** 2022-10-24

**Authors:** Stefan D. Jevtic, Donald M. Arnold, Dimpy Modi, Nikola Ivetic, Anna-Lise Bissola, Ishac Nazy

**Affiliations:** ^1^Department of Medicine, Michael G. DeGroote School of Medicine, McMaster University, Hamilton, ON, Canada; ^2^McMaster Centre for Transfusion Research, McMaster University, Hamilton, ON, Canada; ^3^Canadian Blood Services, Hamilton, ON, Canada; ^4^Department of Biochemistry and Biomedical Sciences, McMaster University, Hamilton, ON, Canada

**Keywords:** COVID-19, platelet, antigen-antibody complex, immune complex, thrombosis, thrombocytopenia, heparin, vaccine

## Abstract

Coronavirus disease 2019 (COVID-19) is a viral respiratory infection caused by the severe acute respiratory syndrome virus (SARS-CoV-2). Vaccines that protect against SARS-CoV-2 infection have been widely employed to reduce the incidence of symptomatic and severe disease. However, adenovirus-based SARS-CoV-2 vaccines can cause a rare, thrombotic disorder termed vaccine-induced immune thrombotic thrombocytopenia (VITT). VITT often develops in the first 5 to 30 days following vaccination and is characterized by thrombocytopenia and thrombosis in unusual locations (e.g., cerebral venous sinus thrombosis). The diagnosis is confirmed by testing for anti-PF4 antibodies, as these antibodies are capable of platelet activation without any cofactor. It can be clinically challenging to differentiate VITT from a similar disorder called heparin-induced thrombocytopenia (HIT), since heparin is commonly used in hospitalized patients. VITT and HIT have similar pathobiology and clinical manifestations but important differences in testing including the need for PF4-enhanced functional assays and the poor reliability of rapid immunoassays for the detection of anti-platelet factor 4 (PF4) antibodies. In this review we summarize the epidemiology of VITT; highlight similarities and differences between HIT and VITT; and provide an update on the clinical diagnosis of VITT.

## Introduction

Vaccine-induced immune thrombotic thrombocytopenia (VITT) is a recently-described hematological disorder that emerged during the mass coronavirus disease 2019 (COVID-19) vaccination programs (1). The incidence of VITT ranges from 1 in 26,500 to 263,000 people after the administration of an adenoviral vector vaccine, with an overall incidence of ~1 in 100,000 (2). VITT is similar to heparin-induced thrombocytopenia (HIT), which is a side effect of heparin exposure, that is also characterized by thrombocytopenia and thrombosis (3, 4). VITT often presents with a severe clinical illness, yet the diagnosis can be missed since it is rare and clinical manifestations are non-specific. Laboratory testing is essential for VITT diagnosis and is based on similar concepts as HIT testing but with important differences. These differences include the type of screening immunoassay used and the need for specialized functional assays.

In this review we provide a pragmatic overview of VITT. We review the differences in clinical presentation between HIT and VITT; VITT pathophysiology; and unique aspects of laboratory investigations.

## Clinical characteristics of VITT

VITT is characterized by new-onset thrombocytopenia and thrombosis following vaccination with an adenoviral COVID-19 vaccine (1, 3). Examples of these include the ChAdOx1 nCoV-19 [AstraZeneca-Oxford] and Ad21.COV2.S [Johnson & Johnson/Janssen] vaccines. VITT patients have a medium time to symptom onset of 15 days post-vaccination (ranging from 7 to 61 days) based on a Canadian cohort of 43 patients (5). The median age of this cohort was 52 years old (ranging from 29 to 73 years old) with no apparent sex-related differences (5). Patients present with thrombocytopenia and the majority have concurrent thrombosis (85.7%) (5). Rarely, patients can present without thrombocytopenia, as occurred in 2/35 patients in the Canadian cohort. Cerebral venous sinus thrombosis (CVST) has been reported as the most common site of thrombosis (36.8%); other locations include deep-vein thrombosis of the lower extremity (23.7%); pulmonary embolism (31.6%); and splanchnic vein thrombosis (19%) (1, 3, 5, 6). Appropriate imaging based on symptom presentation (e.g., MRI venogram for severe headache) should be completed to confirm thrombosis.

A complete blood count, D-dimer, coagulation tests [international normalized ratio (INR), partial thromboplastin time (PTT)], and liver enzymes should be ordered in suspected VITT patients. The median platelet count in VITT ranges from 25,000 to 47,000 based on several reports (3, 7, 8). D-dimer levels are often markedly elevated and may worsen with disease progression (9). If the D-dimer level is <2,000 fibrinogen equivalent units (or four times the upper limit of normal), VITT is unlikely and alternate diagnoses should be considered (10). Coagulation testing (INR, aPTT, fibrinogen) are used to monitor for consumptive coagulopathy that can occur with severe or late presentations. Elevated liver enzymes (aspartate transaminase, alanine transaminase, and alkaline phosphatase) or liver function abnormalities may suggest underlying splanchnic vein thrombosis. While these initial investigations can support the diagnosis, specialized testing is ultimately required for confirmation (see Diagnostic Testing below).

A recent report described eleven VITT patients who presented to hospital with no thrombosis on initial assessment. All eleven patients had thrombocytopenia and severe headache following adenoviral vaccination (4). They also had biochemical features suggestive of VITT (thrombocytopenia, elevated D-dimer) with high anti-PF4/heparin IgG levels confirming the diagnosis. Two patients had CVST and seven did not develop thrombosis. This highlights the fact that VITT may present with isolated thrombocytopenia and, rarely, thrombosis with platelet count levels that are in the normal range. Treatment should not be delayed if clinical suspicion is high (e.g., strongly positive anti-PF4 testing with a high optical density).

## Pathobiology of VITT and HIT

A review of VITT pathobiology is essential to understand the clinical application of specialized screening and diagnostic tests. VITT is a prothrombotic, autoimmune disorder that is a result of pathologic anti-PF4 antibodies (11–13). These antibodies target the cationic protein, PF4, that is normally released from platelets during coagulation (14, 15). Anti-PF4 antibodies bind to PF4 on platelet surfaces to form immune complexes that subsequently activate platelets through the FcγRIIa receptor (16, 17). This results in significant release of intracellular molecules, including serotonin (see Serotonin Release Assay below), with resulting thrombocytopenia and thrombosis. It is postulated that monocytes and neutrophils are also activated, in a similar manner to HIT, but this has yet to be confirmed (18–20).

Similarly, HIT is mediated through anti-PF4 antibodies, but the antibody characteristics and mechanism of platelet activation are different than VITT. HIT occurs following heparin exposure and the immune complex is dependent on this hapten. Heparin, a negatively charged molecule, serves as a scaffold to bridge PF4 molecules. This facilitates anti-PF4/heparin antibody immune complex formation. Heparin binds to a unique site on the PF4 protein that has been well characterized through alanine scanning mutagenesis studies (21, 22). Anti-PF4 antibodies (VITT) bind directly to this heparin-binding site on PF4 and thus do not require heparin as a hapten. Indeed, heparin appears to inhibit VITT anti-PF4 antibody binding to PF4 *in vitro* since they both bind to the same region (22, 23). The trigger for anti-PF4 antibody production is unclear but several reports have speculated that the negatively charged surface of the adenovirus vector or other vaccine components bind PF4 to stimulate antibody production and activation, similar to heparin (13, 24). These distinct molecular differences, including heparin-dependence and anti-PF4 antibody specificity, have important implications for VITT testing.

## Diagnostic testing

### Screening tests-rapid immunoassays and enzyme immunoassays

Screening tests for VITT were initially adapted from HIT assays due to the similarities in pathobiology and antibody specificity. The two broad categories of testing include enzyme immunoassays (EIAs) and rapid assay (25). These immunoassays rely on antibody recognition of PF4, either alone or in complex with a negatively charged scaffold such as heparin (PF4/heparin) or polyvinylsulfate (PVS, PF4/PVS) (26–29). Rapid assays, as the name implies, offer faster turnaround time compared to EIAs and do not require sample batching. For HIT, both the EIA and rapid assays have a high sensitivity (90–99%) but low specificity (~50–70%) for “pathogenic” anti-PF4/heparin antibodies (25, 30–36). Reduced specificity is due to false positive results from patients with “non-pathogenic” anti-PF4/heparin antibodies. These “non-pathogenic” forms are unable to bind the appropriate site on PF4 required for immune complex formation and subsequent platelet activation and thus do not manifest with HIT (21, 35). Although rapid assays have similar efficacy in HIT testing, this does not hold true in the context of VITT, as will be discussed below.

EIAs quantify the optical density emitted from anti-PF4/heparin antibodies in patient serum when mixed with a secondary antibody. They consist of microtiter plates containing antigen complexes (PF4/heparin) bound to the well surface. Patient sera is added to the well and anti-PF4/heparin antibodies bind to the antigen. A secondary antibody (anti-human IgG) that can emit light is added and the degree of transmission is quantified. Commercially available EIAs for HIT detect either all immunoglobulin classes (polyspecific, i.e., IgG/A/M) or IgG-only antibodies. When used for VITT testing, commercial EIA has been reported to have a sensitivity of 100% and the specificity of commercial, in-house, and anti-P4F/heparin ranging between 95.6 and 97.4% (5). Various rapid assays are commercially available for the diagnosis of HIT including the LIA, chemiluminescence immunoassay (CLIA), particle gel immunoassay (PaGIA), and lateral flow assay (LFA). The LIA measures light transmission of a sample containing PF4/PVS-coated latex nanoparticles mixed with a HIT-mimicking antibody (30, 31). This antibody agglutinates the nanoparticles, thus resulting in low light transmission through the sample. When patient plasma containing anti-PF4/heparin antibodies is added, there is competition for binding to the nanoparticles. This disrupts the agglutination and results in higher light transmission. Therefore, the amount of patient anti-PF4/heparin antibodies is directly related to the degree of light transmission. The CLIA also utilizes PF4/PVS coated particles to capture anti-PF4/heparin antibodies and then measures the emitted light of an isoluminol-labeled anti-human IgG secondary antibody (25). Similarly, the ID-PaGIA measures the ability of patient sera to agglutinate beads coated with PF4/heparin complexes (32). Lastly, LFAs capture antibodies bound to PF4/PVS when as patient samples migrate with buffer across a test strip, appearing as a visible colored line when positive (37). Rapid assays thus represent an attractive option for HIT testing because of their ease of use, rapid turnaround time (<1 h), and superior performance compared to standard EIAs (slightly lower sensitivity, but greater specificity) (30, 33). However, rapid assays should not be used for VITT testing due to their inferior sensitivity (high false-positive rate) compared to EIA (12, 37).

Platton et al. (11) tested 23 suspected or probable VITT patients using six commercially available EIAs and four rapid assays. IgG-specific EIAs had comparable diagnostic performances to the polyspecific EIAs, with 5/6 tests showing >90% sensitivity. On the other hand, the rapid tests performed poorly in VITT: three of the four rapid assays had diagnostic sensitivities between 0 and 5.9% while the fourth had a sensitivity of 45.5% (13). Another study also found similarly poor detection of anti-PF4 antibodies in VITT patients with the following assays: ID-PaGIA (3/12, 25% detected), CLIA/HemosIL AcuStar HIT-IgG (8%), and LFA/Milenia QuickLine HIT-Test (0%) (37). Using the same samples, two commercial EIAs (ZYMUTEST HIA IgG-EIA and Immucor PF4 IgG-EIA) were shown to better identify anti-PF4 antibodies in 11/12 (92%) and 12/12 (100%) VITT patients. The superiority of EIAs was confirmed in another study of 9 VITT patients that showed reduced accuracy of rapid assays for VITT antibodies (12). All rapid assays tested in this study were negative for anti-PF4 antibodies whereas the three EIAs used showed variable results depending on the antigen target. Only the PF4-PVS EIA (Lifecodes PF4 IgG, Immucor) successfully identified high titres of anti-PF4 antibodies in 7/9 patients. The PF4/heparin EIA (Asserachrom HPIA, Stago) identified anti-PF4 antibodies in 5/9 suspected patients while the platelet-lysate/heparin EIA (Zymutest HIA IgG, Hyphen) only identified antibodies in 4/9 suspected patients. Finally, a multi-center UK study evaluating anti-PF4 EIAs and rapid assays showed rapid assays to have inferior performance compared to EIAs for identifying anti-PF4 VITT antibodies (38).

Therefore, while rapid assays are often used to rule-out the presence of anti-PF4/heparin antibodies in HIT, they perform poorly in VITT. EIAs are the preferred screening assays as they can accurately identify anti-PF4 VITT antibodies. The Lifecodes Immucor PF4-EIA has demonstrated the best performance in this regard. Reference laboratories should be able to perform more than one type of EIA, given that no single assay detects all VITT sera. Future work should look to evaluate the diagnostic capabilities of EIAs to further substantiate their usefulness as a diagnostic test for VITT due to the limited sample size of these studies. However, functional platelet activating assays remain the gold-standard test for laboratory confirmation of VITT.

### Functional assays (HIPA/PEA/SRA)

Functional platelet activation assays are required to confirm the diagnosis of HIT after a positive screen for anti-PF4/heparin antibodies with the EIA or rapid assays (39). Functional assays thus reduce false positive results due to non-pathogenic antibodies (34, 40). Examples of HIT functional assays include the heparin-induced platelet aggregation assay (HIPA), heparin-induced multiple electrode aggregometry (HIMEA), PF4-dependent P-selectin expression assay (PEA), and the serotonin release assay (SRA) (39, 41–43). The HIPA, PEA and SRA rely on the use of fresh donor platelets that are mixed with patient serum, while the HIMEA is a whole blood-based assay (see Table 1). In the case of the HIPA, HIEMA and SRA, varying exogenous heparin doses are used to confirm heparin-dependence or independence. The assays mostly differ in their measured endpoints of platelet activation: HIPA measures platelet aggregation *via* turbidity change (assessed visually), while the HIMEA measures aggregation by electrical impedance; the PEA measures P-selectin expression on the platelet surface; and the SRA measures radioactive serotonin release from platelet granules (34, 39, 41–43, 47). These assays can also be used to identify HIT patients who may safely receive heparin products following treatment. For example, in a HIT patient treated with intravenous immunoglobulin, the SRA (functional) test will be negative despite a persistently positive EIA (48).

**Table 1:** Summary of platelet functional assays in HIT and VITT.

**HIT**		**VITT analog**		
**Assay**	**Methodology**	**Assay**	**Difference in methodology**	**Reported results for clinical VITT**
Serotonin release assay (SRA)	• Measures release of radioactive serotonin from activated platelets • Washed platelets treated with heparin and patient sera	PF4-SRA	Modified SRA with no heparin and washed platelets preincubated with PF4	3/3 (44) 5/5 (22)
Heparin-induced platelet activation assay (HIPA)	• Visual detection of platelet aggregation • Washed platelets treated with heparin and patient sera	PIPA	Modified HIPA with no heparin and washed platelets preincubated with PF4	28/28 (1)
Heparin-induced multiple electrode Aggregometry (HIMEA)	• Platelet aggregation measured by changes in electrical impedance. • Whole blood mixed with heparin and patient sera	HEMA	Modified HEMA with no heparin	4/5 ^*^(45) 50/52 ^**^(8)
PF4-dependent P-selectin expression assay (PEA)	• Expression of P-selectin *via* flow cytometry • Washed platelets treated with patient sera and PF4	PIFPA^***^	Expression of P-selectin *via* flow cytometry Whole blood assay with PF4 preincubation before sera addition	16/16 (46)

* Negative sample was ruled inconclusive.

** Negative samples were patients undergoing IVIG.

*** Closest analogue. All samples tested were positive for anti-PF4 antibodies.

Functional HIT assays were quickly implemented for VITT testing once it was discovered that patients had anti-PF4 antibodies (45). However, standard functional assays often produced false-negative results in VITT. Therefore, a modification of the functional assay is needed by adding exogenous PF4 instead of heparin (1, 22, 44). In classic HIT, exogenous heparin serves as a scaffold to complex PF4 and induce a conformational change such that it can be recognized by anti-PF4/heparin HIT antibodies (40). This results in a “heparin-dependent” platelet activation, as evidenced in the SRA (39, 44). In contrast, heparin was found to *reduce* the sensitivity of platelet activation assays in VITT and produced a false-negative SRA. However, the addition of exogenous PF4 significantly increased the sensitivity of HIT assays and produced an activation pattern similar to HIT. The heparin-mediated inhibition (at pharmacological concentrations, i.e., 0.1 to 0.5 IU/mL heparin) of platelet activation in VITT can be explained by the recent finding that the majority of anti-PF4 VITT antibodies bind to the heparin-binding site on PF4 (22). Therefore, heparin should be avoided in VITT functional platelet testing and exogenous PF4 should be added (49). In one study, the addition of heparin to the PF4-enhanced HIPA assay reduced the sensitivity from 100 to 20% of clinically diagnosed VITT samples (5). In another study, sensitivity was reduced from 100 to 46.2% when heparin was added to the PF4-enhanced SRA (1). Interestingly, both the PEA and the PF4-induced flow cytometry–based platelet activation assay (PIFPA) already require PF4 and do not use heparin in their reactions (46). Both assays measure P-selectin expression *via* flow cytometry for their activation endpoints; the main differences between the PFIPA and PEA is the use of a whole blood-based assay compared to washed platelet-based assay, respectively.

PF4-enhanced functional assays can confirm whether anti-PF4 antibodies are capable of platelet activation and whether persistent anti-PF4 antibodies continue to be pathogenic. This was highlighted in a recent study of 69 patients with thrombocytopenia and thrombosis syndrome (TTS, an epidemiologic classification that includes VITT) (8). The sensitivity and specificity of functional platelet assays in this cohort were 96 and 78%, respectively. The two patients who yielded false negative platelet activation had receive intravenous immunoglobulin prior to blood collection, which is known to interfere with these assays (44). This suggests that functional assays may even approach 100% sensitivity if appropriate clinical context and pre-analytical variables are used, and with optimal assay reactivity. Rarely, PF4 supplementation may be required as demonstrated by “SRA-negative HIT” patient sera (47, 50). In one study, four of eight VITT patients (50%) with thrombocytopenia but without thrombosis also tested strongly positive for anti-PF4, platelet-activating antibodies. Functional assays can thus confirm the diagnosis in patients with atypical VITT presentation (e.g., thrombocytopenia alone on initial presentation). However, given that the excellent operating characteristics of the EIAs, functional assays are not required to confirm the diagnosis of VITT. This contrasts with HIT where the screening test has a low specificity and confirmatory functional assays are needed to confirm the diagnosis.

Functional assays can also potentially identify disease resolution in the context of persistent anti-PF4 antibodies following VITT. In a longitudinal study of 35 VITT patients, 32 (91%) continued to test positive on anti-PF4/heparin IgG EIA, albeit with significantly reduced optical density results (51). In those who had follow-up >12 weeks, the majority (14/15, 93%) became negative in the platelet-activation assays. The remaining patient exhibited recurrent thrombocytopenia with persistently high-titre anti-PF4/heparin IgG and positive platelet-activation. Therefore, platelet functional assays may be used to monitor disease activity longitudinally in patients with ongoing symptoms or evidence of disease recurrence. It is still unclear whether persistent EIA positivity indicates potential for recurrent disease.

VITT functional assays are thus a useful adjunct and diagnostic tool for patient cases that are atypical or require longitudinal monitoring. When testing well clinically defined VITT patient samples, VITT functional assays have reliable and accurate detection. Neither the PF4 serotonin release assay (PF4- SRA), the PF4-induced platelet activation assay (PIPA), or flow-cytometry based assay (PIFPA) missed any of the clinically diagnosed VITT samples, whereas the HEMA missed three samples in two different studies. Of these three samples, one was inconclusive while the other two were patients undergoing IVIG treatment (Table 1) (1, 8, 44–46). Given the relative novelty of VITT and rapidity at which these assays were developed, the majority of studies featured small, well-selected patient populations. No significant large-scale comparative studies have been done to compare the functional assays to each other as of this publication. It is because of this small sample population that the sensitivity and specificity of these tests has not been well defined; however, by all initial indications the assays appear to be strong diagnostic tools for VITT and should be considered when EIA testing is not available or additional supportive testing is required.

## Discussion

VITT is a prothrombotic disorder that has been characterized through application of existing knowledge related to HIT. Although these disorders share many similarities, clinicians should be aware of several important differences: First, HIT antibodies and VITT antibodies have different binding sites on PF4. Second, anti-PF4/heparin EIAs have high sensitivity and high specificity for the VITT diagnosis, whereas the specificity is low for HIT. Third, rapid diagnostic assays developed for HIT often yield false-negative results for VITT and should not be used as a diagnostic test for VITT.

## Author contributions

SJ, DA, DM, NI, A-LB, and IN were all involved in the conceptualization, writing, and editing of the manuscript. All authors contributed to the article and approved the submitted version.

## Funding

Funding was received from the Canadian Institutes of Health Research (CIHR 452655) awarded to IN.

## Conflict of interest

The authors declare that the research was conducted in the absence of any commercial or financial relationships that could be construed as a potential conflict of interest.

## Publisher's note

All claims expressed in this article are solely those of the authors and do not necessarily represent those of their affiliated organizations, or those of the publisher, the editors and the reviewers. Any product that may be evaluated in this article, or claim that may be made by its manufacturer, is not guaranteed or endorsed by the publisher.
